# Supplementary material for: Relationship of preoperative oral hypofunction with prognostic nutritional index in gastric cancer: A case-control retrospective study
Source: PLoS One. 2023 Jun 1;18(6):e0283911. doi: 10.1371/journal.pone.0283911 (PMC10234551; doi:10.1371/journal.pone.0283911)
Supplement: Attachment — Submitted filename: Response to Reviewers.docx [file pone.0283911.s001.docx]

March 20, 2023

Gaetano Isola, Ph.D.

Editor-in-Chief

*PLOS ONE*

Dear Editor:

Thank you for your decision letter and for giving us the opportunity to revise our manuscript (PONE-D-22-23725). We wish to re-submit entitled “**Relationship of preoperative oral hypofunction with prognostic nutritional index in gastric cancer: a case-control retrospective study**.”

We thank you and the reviewers for your thoughtful suggestions and insights. The manuscript has benefited from these insightful suggestions. I look forward to working with you and the reviewers to move this manuscript closer to publication in *PLOS ONE*.

We have reviewed all editorial and technical suggestions provided in the decision letter and made changes to our manuscript accordingly. We are hereby resubmitting a revised manuscript conforming to all of the editor’s and reviewers’ comments. In particular, we have addressed all the comments in a point-by-point manner and revisions are indicated highlighted in the revised manuscript.

We hope that the revised manuscript is now suitable for publication in your journal. Thank you for your consideration. We look forward to hearing from you.

Sincerely,

Dr. Atsushi Nakayama

The First Department of Oral and Maxillofacial Surgery, School of Dentistry, Aichi-Gakuin University, Nagoya, Aichi Prefecture, Japan

E-mail: nkym8020@dpc.agu.ac.jp

**Editor Comments:**

Journal Requirements:

When submitting your revision, we need you to address these additional requirements.

1. Please ensure that your manuscript meets PLOS ONE's style requirements, including those for file naming.The PLOS ONE style templates can be found at　https://journals.plos.org/plosone/s/file?id=wjVg/PLOSOne_formatting_sample_main_body.pdf and　<https://journals.plos.org/plosone/s/file?id=ba62/PLOSOne_formatting_sample_title_authors_affiliations.pdf>

**Response:** We would like to thank the Editor for evaluating our manuscript and for the constructive comments. The reviewers have indicated that the manuscript meets the style requirements (including the requirement regarding file names). We have revised the manuscript to conform to the style template.

2.In your Data Availability statement, you have not specified where the minimal data set underlying the results described in your manuscript can be found.PLOS defines a study's minimal data set as the underlying data used to reach the conclusions drawn in the manuscript and any additional data required to replicate the reported study findings in their entirety.All PLOS journals require that the minimal data set be made fully available.For more information about our data policy, please see http://journals.plos.org/plosone/s/data-availability.Upon re-submitting your revised manuscript, please upload your study's minimal underlying data set as either Supporting Information files or to a stable, public repository and include the relevant URLs, DOIs, or accession numbers within your revised cover letter.For a list of acceptable repositories, please see http://journals.plos.org/plosone/s/data-availability#loc-recommended-repositories.Any potentially identifying patient information must be fully anonymized.

Important:If there are ethical or legal restrictions to sharing your data publicly, please explain these restrictions in detail.Please see our guidelines for more information on what we consider unacceptable restrictions to publicly sharing (data:http://journals.plos.org/plosone/s/data-availability#loc-unacceptable-data-access-restrictions.Note that it is not acceptable for the authors to be the sole named individuals responsible for ensuring data access.We will update your Data Availability statement to reflect the information you provide in your cover letter.

Additional Editor Comments:

Please revise in accordance to both reviewers before any further comments.

**Response:** We apologize for not providing a Data Availability statement. We have referred to the details provided above and in the journal guidelines and have included a Data Availability statement in the revised manuscript. (Page 30, Line 461-467)

**Reviewer: 1**

Comments to the Author

The authors aimed to elucidate the clinical relevance of prognostic nutritional index (PNI) for assessing oral function in patients with gastric cancer. As described in the manuscript, there have been few studies concurrently examining preoperative nutritional status and oral function decline in gastric cancer cases so far.Therefore, the concept of this study is unique and potentially of interest of oncological care-givers.Unfortunately, however, there are some flaws spoiling its scientific significance.

**Response**: The authors would like to thank Reviewer 1 for your time and efforts in reviewing our manuscript and for providing comments, which have considerably helped us improve our manuscript. We have made revisions based on your comments and have provided our point-by-point responses below. The revisions are indicated in yellow highlighted text in the revised manuscript. We hope that our responses and revisions appropriately address your comments.

1)Relationship between PNI and several oral function factors were investigated and only togue pressure showed statistically significant association.

It appeared to be a merely "chanced" event owing to a small size of the cohort.

**Response:** We acknowledge the need to consider the small sample size of our study. However, we were unable to address this issue in this research. This study was conducted as an exploratory study. We realize that our original explanation was unclear and have therefore, revised the relevant text in the manuscript as follows:

“As this study was conducted as an exploratory research to formulate a hypothesis and give the small sample size, the relationship between the PNI and tongue pressure could not be determined clearly.” (Page 21, Lines 303-306）

2) Use of univariate significance to produce multivariable models denies confounding and Simpson's paradox. Please eschew this and produce a proper model showing the independent value of these markers over and above known clinical features after adjustment.

**Response:** Based on your comment, we performed the analysis again, adjusting for known clinical features. The results are presented in Table 3. Analysis including existing clinical factors, CRP and tongue pressure were significant

3) Dichotomous categorization using cut-off values, even though being a common method in clinical research, can produce several problems (BMJ 2006;332:1080) (Stat Med 2006;25:127-41).Dividing the patients into two groups using an arbitrary cut-off value leads to decrease in statistical power and incomplete correction for confounding factors.Employing data-derived cut-points can cause serious bias and incline to unrealistic scenario.This point should be mentioned as a potential limitation.

**Response:** Please note that we have added a new part in the manuscript to address your comments and have cited new references (References #41, 42). The added part is as follows:

“In epidemiological studies, dichotomous categories are used less frequently, and the use of continuous variables or multiple categories is preferred [41,42]. The use of multiple categories and creating ordinal variables is preferable to dichotomization. Therefore, a large prospective validation study is warranted to confirm these results.” (Lines 297–301)

4)As the authors cited [22, 23], PNI has some definitional variations.Their definition of PNI is not Buzby's, but Onodera's.This should be explicitly indicated.

**Response:** Based on your suggestion, we have provided this information in the revised manuscript as follows:

“In this study, Onodera's method, which is a simple formula, was used to calculate the PNI.” (Lines 99–100)

5) The Discussion section is too long.It should not be a redundant bibliographical review. Please make it more succinct and to the point.

**Response:** Based on your comment regarding the lack of clarity and the length of the Discussion section, we have revised the section accordingly.

6) In page 6, the authors documented "all blood specimens were collected at least one month prior to the preoperative examination for gastric cancer surgery".

Maybe, "at most" is better.

**Response:** Based on your comment, the notation has been changed as follows:

“All blood specimens were collected at most 1 month prior to the preoperative examination for gastric cancer surgery.” (Lines 96–97)

**Reviewer: 2**

Comments to the Author

It is an interesting topic.We always contribute the malnutrition in patients with gastric cancer to the disease itself, and never associate it with oral hypofunction.In this study, the authors concluded that oral function is decreased in perioperative patients with gastric cancer, and decreased tongue pressure is associated with decreased prognostic nutritional index, which may help to improve the management of perioperatvie nutrition in patients with gastric cancer.Through comprehensively read this manuscript, I found some points need revision and listed them as follows:

**Response:** The authors would like to thank Reviewer 2 for your time and efforts in reviewing our manuscript and for providing comments, which have considerably helped us improve our manuscript. We have made revisions based on your comments and have provided our point-by-point responses below. The revisions are indicated in yellow highlighted text in the revised manuscript. We hope that our responses and revisions appropriately address your comments.

1) A cross-sectional study is a type of observational research that analyzes data of variables collected at one given point in time across a sample population or a predefined subset.Therefore, strictly speaking, this study is not a cross-sectional study but a case-control retrospective study.

**Response:** We apologize for the overlook. We have revised the relevant text about the study design as follows:

“No *a priori* sample size calculation was required, as this was a case-control retrospective study.” (Lines 81–82)

2) Why patients with an aborted surgery were excluded, did it affect oral function?

**Response:** We realized that our explanation was unclear. We believe that selection bias is an issue in this study. In contrast, regarding the exclusion criteria for this study, we believe that the patients for whom surgery was discontinued were duplicate cancer cases with oral cancer and that selection bias due to exclusion is less likely. This point was noted in the limitation.(Page 21,Line 307-309)

3) In the methods section, the authors mentioned that 17 patients with missing data and were excluded.However, according to Fig,1, all 17 patients were excluded because of the previous 5 exclusion criteria, but not missing data.

**Response:** Based on your comment, we have revised Fig 1 accordingly. Corrected an error in the figures.

4) Why all blood specimens were collected at least one month prior to the preoperative examination for gastric cancer surgery? Is one month delay from blood test to surgery too long?

**Response:** We apologize for the overlooks in the “Data analysis” subsection. Blood samples were collected within 1 month before surgery. To address your comment, we have provided this information in the revised manuscript as follows:

“All blood specimens were collected at most 1 month prior to the preoperative examination for gastric cancer surgery.” (Lines 96–97)

5) Fisher's exact test is not a method to test normally distributed data, which should be tested by t test or ANOVA.Fisher's exact test is used to test categorical variable with sample smaller than 40. In table 2, all variables are transformed to be categorical variable, therefore, only chi squared test will be used in the analysis, thus normality assessment is also not necessary.

**Response:** We would like to thank the reviewer for his/her comment concerning statistical analysis and for pointing out this error. We used the t-test or ANOVA in the study. We have provided this information in the revised manuscript and in Table 2. The added part is as follows:

“Univariate analysis was performed to examine the association between the PNI and oral function. Next, multivariate analysis was performed using selected prognosis-related factors. Patient characteristics and their relationship to the PNI were analyzed using t-test or ANOVA.” (Lines 148–151)

6) A PNI of 40 or less was used as the objective variable in the data analysis section, this cut-off is also applied in Table 1, however, in results, the patients were divided by the cut-off 45, why?

**Response:** In this study, the cutoff value for PNI was set at 40 based on previous studies:

“Patients with PNI <40 (24 patients, 25.3%) were classified into the low PNI group and those with PNI >40 (71 patients, 74.7%) were classified into the high PNI group [30].” (Lines 169–170).

7) As a retrospective study, the authors should explain why these patients underwent an oral function examination, which is not a routine preoperative test of gastric cancer patients.

**Response:** Please note that we have provided this information in the revised manuscript as follows:

“Preoperative oral examination was performed to reduce the risk of pneumonia, wound infection, intraoperative tooth loss, and various complications related to the oral region. These parameters were selected for evaluation, as oral bacteria and oral functions, such as chewing, swallowing, and tongue movement functions, are strongly related to these complications.” (Lines 109–112)

8) In the results section, the number of patients with oral dysfunction was presented, however, as sample sizes were not the same between two group, such presentation can not show the differences between groups, therefore, percentage should also be listed.

**Response:** Based on your suggestion, we have provided the percentages in the Results section and in Table 2.

9) The writing of the manuscript should be extensively checked, for instance, "postoperative surgical complications" is expressed repeatitively, "postoperative complications" is OK.

**Response:** We apologize for the errors. The terminology has been unified with postoperative complications.
