# Supplementary material for: Relationship of preoperative oral hypofunction with prognostic nutritional index in gastric cancer: A case-control retrospective study
Source: PLoS One. 2023 Jun 1;18(6):e0283911. doi: 10.1371/journal.pone.0283911 (PMC10234551; doi:10.1371/journal.pone.0283911)
Supplement: Attachment — Submitted filename: Response_to_Reviewers.docx [file pone.0283911.s002.docx]

**Reviewer(s)' Comments to Author:**

**Reviewer #1:**

Dear authors,
I revised the article “Relationship of preoperative oral hypofunction with prognostic nutritional index in gastric cancer: a case-control retrospective study”. The goal of this research was to investigate the relationship between the changes in preoperative oral function and the PNI, an immunonutritional index, in patients with gastric cancer, since a decline in preoperative oral function may contribute to postoperative complications. Furthermore, we investigated the correlation between PNI and the seven factors related to oral hypofunction.

The study is interesting and relevant, which enrolled 112 patients diagnosed with primary gastric cancer.

I endorse the publication. The study is straightforward and correct.

**Reviewer #2:**

In the manuscript entitled: "Relationship of preoperative oral hypofunction with prognostic nutritional index in gastric cancer: a case-control retrospective study" the authors recruited patients with perioperative gastric cancer and examined preoperative oral hypofunction and its relationship with prognostic nutritional index. This case-control study analyzed 95 patients who underwent oral function management.
The authors found that significant differences in C-reactive protein, neutrophils, and tongue pressure (p<0.01) between the two groups. However, oral hygiene, oral dryness, occlusal force, tongue–lip motor function, masticatory function, and swallowing function were not significantly different. Multivariate analysis showed that C-reactive protein (p<0.01) and tongue pressure (p<0.05) were independent risk factors for oral hypofunction.
The authors concluded that oral function decreased in perioperative patients with gastric cancer, and decreased tongue pressure is associated with decreased prognostic nutritional index.

Major comments:
In general, the idea and innovation of this study regards the analysis of periodontitis and oral hypofunctions is interesting and novel because the role these aspects in medicine are validated but further studies on this topic could be an innovative issue in this field could be open a creative matter of debate in literature by adding new information. Moreover, there are few reports in the literature that studied this interesting topic with this kind of study design.
The study was well conducted by the authors; However, there are some concerns to revise that are described below.
The introduction section resumes the existing knowledge regarding the important factor linked with periodontitis and related biomarkers.
However, as the importance of the topic, the reviewer strongly recommends, before a further re-evaluation of the manuscript, to update the literature through read, discuss and must cites in the references with great attention all of those recent interesting articles, that helps the authors to better introduce and discuss the role of some early biomarkers of periodontitis (TGF beta 1, circulating cells) which could better early diagnose the risk of both periodontitis and implant restoration especially in patients with periodontitis.

**Response:** We have cited three references to support the explanation on page 4, lines 47-51.

The authors should be better specified, at the end of the introduction section, the rationale of the study and the aim of the study.

**Response:** Thank you for the valuable comment. We have modified the manuscript accordingly (page 5, lines 73-74)

In the central section, should better clarify inclusions and exclusions criteria of the selected sample.

**Response:** We apologize for the unclar inclusion and exclusions criteria. We have revised the manuscript accordingly (page 6, lines 80-86).

The discussion section appears well organized with the relevant paper that support the conclusions, even if the authors should better discuss the relationship regarding the role exerted by relevant biomarkers of periodontitis in people who had gastric cancer and request also implant restoration.

**Response:** Thank you for pointing out this comment. We have added an explanation to pages 22-23, Lines 311-321.

The conclusion should reinforce in light of the discussions.In conclusion, I am sure that the authors are fine clinicians who achieve very nice results with their adopted protocol. However, this study, in my view does not in its current form satisfy a very high scientific requirement for publication in this journal and requests a revision before a futher re-evaluation of the manuscript.

Minor Comments:

Abstract:
- Better formulate the abstract section by better describing the aim of the study

**Response:** Thank you for the valuable comment, we have revised the manuscript accordingly. (Page 3, lines 24-26)

Introduction:
- Please refer to major comments

Discussion
- Please add a specific sentence that clarifies the results obtained in the first part of the discussion

**Response:** We realize that our original explanation was unclear. We have added the the meaning of the results and their implications. (Page 18, 222-223)
